# Body maps of the sensation of musical groove

**DOI:** 10.1093/pnasnexus/pgaf306

**Published:** 2025-09-24

**Authors:** Maria A G Witek, Tomas E Matthews, Toni A Bechtold, Virginia Penhune

**Affiliations:** Department of Music, School of Languages, Cultures, Art History and Music, University of Birmingham, Edgbaston, Birmingham B15 2TT, United Kingdom; Center for Music in the Brain, Aarhus University, Aarhus 8000, Denmark; Department of Music, School of Languages, Cultures, Art History and Music, University of Birmingham, Edgbaston, Birmingham B15 2TT, United Kingdom; Lucerne School of Music, Lucerne University of Applied Sciences and Arts, Lucerne-Kriens CH-6010, Switzerland; Department of Psychology, Concordia University, Montreal H3G 1M8, Canada

**Keywords:** music, rhythm, groove, emotion, embodiment

## Abstract

Rhythmic music often leads to an urge to move the body in time with the music. This urge to move can be a pleasurable experience. In psychology, we define the pleasurable wanting to move to music as groove. Here, we investigate where in the body these two groove components—movement and pleasure—are felt and whether the embodied sensations depend on the musical genre. Using a body sensation map paradigm, we found that the funk genre, which elicited high levels of groove, increased sensations across the whole body, including in the head, shoulders, upper chest, abdomen, arms, hands, hips, legs, and feet. Importantly, wanting to move and pleasure produced distinct body maps, with wanting to move associated with more sensation in the extremities and pleasure more associated with feelings in the chest and abdomen. Exploratory analyses also found an inverted U-shaped relationship between wanting to move and pleasure ratings and the rhythmic complexity of the excerpts, as indexed by pulse entropy, and that medium pulse entropy produced sensations in the upper chest, shoulders, hips, and ankles. The results are discussed in relation to theories of embodied predictive processing, highlighting the potential role of interoception in musical prediction and reward. Overall, our study shows clear patterns of embodied differentiation for different components and levels of groove.

Significance Statement Music is an important source of pleasure in people's lives. Here, we show that groove—the pleasurable urge to move to music—can be localized in the body, with wanting to move associated with the extremities and pleasure with the chest and abdomen. Body sensations increased when listening to funk music and were modulated by rhythmic complexity. Because rhythmic complexity is associated with predictability, our results suggest a role for interoception and somatosensory feedback in predictive processing. This provides important evidence for the central role of the body in musical experience and in the human mind more broadly.

## Introduction

While all musical emotions can be considered embodied (1), the effects of musical groove on the body are particularly palpable (2). Psychological research often operationalizes groove according to two components—wanting to move and pleasure (3, 4)—but there are also important social, immersive, and cultural components to the phenomenon (5, 6). Beyond its appeal to overt movement, groove is also described as an embodied feeling (7, 8). But where in the body is groove felt? And do bodily sensations change as a function of the type and rhythmic structure of the music?

The embodiment of emotion has been linked to a number of processes (9), including the perception of the body's internal state changing as a result of homeostatic equilibration following physiological arousal (10, 11). Researchers have documented where such changes are felt using a bodily sensation map (BSM) paradigm developed by Nummenmaa et al. (12). They found that emotions, as induced by words, stories, movies, and facial expressions, can be differentiated by where in the body they are felt, as indicated by coloring on a 2D body silhouette. These BSMs appear to be consistent across cultures, suggesting that they may be universal (13), mirroring the more widely known universality of emotional facial expressions (14). The data from BSM studies highlight the importance of the somatosensory system and interoception in the subjective experiences of emotion (15).

BSMs have been recorded for music as well (16). Across both Western Europeans and East Asians, happy and danceable songs increased sensations in the arms, legs, and head, while sad, tender, and scary songs increased sensations in the chest and head. Aggressive songs were associated with sensations in the head. These sensation patterns also corresponded with certain structural features extracted from the music, including timbre, tonality, rhythm, and dynamics (16). Structural features of music have also been associated with overt movements of certain body parts. Burger et al. (17) found that a number of rhythmic features, such as pulse clarity, percussiveness, and spectral flux, positively correlated with the speed of movements in the body's center of mass, head, hands, and feet, with some features also producing more movement overall.

Focusing more specifically on musical structure, Daikoku et al. (18) recorded BSMs in response to chord progressions, showing that body sensations depended on the interaction of structural uncertainty and surprise, specifically in the head, abdomen, chest, and heart region, which correlated with ratings of aesthetic appreciation. The authors suggest that prediction—a key mechanism for the experience of emotion in music (19)—might be associated with specific visceral sensations in the chest and abdomen.

Prediction is also central to groove and is thought to be the mechanism that explains why certain kinds of rhythms elicit the pleasurable desire to move to the beat (20–22). There is an inverted U-shaped relationship between rhythmic complexity and groove (23–26), purportedly because intermediate levels of complexity create a balance between predictability and uncertainty via strongly weighted prediction errors that invite moving to the beat to reinforce the underlying meter (i.e. the predictive model) (20, 21).

A common approach to measuring rhythmic complexity in real music is to estimate its pulse clarity, which indexes the degree to which listeners can perceive the underlying beat (27). Manipulating pulse clarity affects the ability to synchronize movements to music (28). Furthermore, pulse clarity is correlated with the tap timing of expert musicians (29). Here, we use an inverse measure of pulse clarity—pulse entropy (30)—which indexes the degree to which there is an identifiable beat and how variable or certain the timing of that beat is. Low pulse entropy music has a clear and certain beat, while high-pulse entropy music has an unclear and uncertain beat. Like other kinds of rhythmic complexity, pulse entropy has been shown to have an inverted U-shaped relation with ratings of wanting to move and pleasure (31). Importantly, beat timing uncertainty is directly (inversely) related to rhythmic predictability, and hence, measuring effects of pulse entropy gives us a way of exploring body sensations in relation to temporal predictability in groove.

Aside from levels of complexity, groove is also associated with certain musical genres. Funk is almost synonymous with groove (7), reflecting its strong association with a pleasurable desire to move to music. Certain kinds of jazz are also strongly associated with groove (8). Other rhythm-based musical genres, such as rock, may still elicit groove sensations but is not as strongly groove-inducing as funk and jazz (32). The extent to which a track from a given genre will elicit a pleasurable desire to move may depend on the specific musical structure of that track, including its level of complexity (32, 33), but there may also be nonstructural dimensions that drive the groove response, such as feelings of immersion (5) and sociocultural expectations (6).

Groove does not merely elicit a desire to move; it has also been measured as producing more synchronized movement (34, 35). Music that can be described as groove oriented is also harder to resist moving to (36). It activates the motor system of the brain, including both cortical and subcortical motor areas (37, 38). Interestingly, it appears that groove ratings are more closely related to how well participants perceive themselves to synchronize to music rather than their actual synchrony performance, suggesting there is something about the feeling of moving that is important to the groove experience (39).

The feeling of moving to music is pleasurable (40). However, the embodied nature of pleasure in groove has been harder to study, as it is not as easily measurable as overt body movements. Arousal, a key element of pleasure, has been measured via pupillometry, with groove associated with increased pupil dilation (41) and pupil size drift rate (42). Groove is also associated with activation of the reward network in the brain, especially when intermediately complex rhythms are combined with intermediately complex chords (25).

There has been some debate about whether the movement and pleasure components of groove are separable or if they are part of a single construct. These two measures normally correlate strongly (43), and it is not clear whether they are causally linked and, if so, in what direction. Studies on musical anhedonics—i.e. people whose pleasure from music is attenuated or absent (44)—showed that anhedonics still experience the pleasurable urge to move to music, suggesting that movement might be a protected route to pleasure from music in this group (43, 45). Locating wanting to move and pleasure as sensations in the body might aid in distinguishing these two components of groove. Previous BSM research suggests that emotional valence is more centralized in the body, being felt in the abdomen and chest (16, 18), and that danceable music is associated with feelings in the extremities (16). It may be that these body areas also differentiate the movement and pleasure components of embodied feelings of groove.

In this study, we used the BSM paradigm (12) to measure where in the body people feel groove. We tested two groove components—wanting to move and pleasure—since these components seem more easily localized in the body compared with other components of groove, such as immersion and sociality. We recorded subjective ratings and measured body sensations of these two groove components as induced by different genres. As an additional exploratory analysis, we tested effects of the music's beat timing uncertainty (and hence its predictability) on groove component ratings and body sensations by measuring the music's pulse entropy, representing the rhythmic complexity of the music and the predictability of its beat. We hypothesized that

1. Funk will elicit higher wanting-to-move and pleasure ratings compared with jazz funk and rock.
2. Funk will be associated with stronger body sensations in the chest and abdomen area and the extremities.
3. The movement and pleasure components of groove can be distinguished by BSMs, with movement being more associated with sensations in the extremities and pleasure more associated with sensations in the chest and abdomen.
4. There will be an inverted U-shaped relationship between pulse entropy and ratings of both wanting to move and pleasure.
5. Intermediate levels of pulse entropy will be associated with stronger sensations in both the chest and abdomen area and the extremities.

We used real musical stimuli that would likely be unfamiliar to participants and recorded perceived familiarity for each track to be sure. Years of musical training were recorded, as this has previously been shown to affect groove responses (25, 37). We also asked participants to report genre preferences to check that there was no imbalance in the general liking for the music used in the experiment.

## Materials and methods

### Participants

One hundred and eight participants were recruited via Prolific, including 58 men, 48 women, and two nonbinary. The mean age was 27.9 (SD = 8.39). Participants were from Europe, Africa, and America. The average years of musical training was 1.94 (SD = 3.99) for formal and 2.43 (SD = 3.95) for informal training (i.e. self-taught). Ninety-two participants were right handed and 16 were left handed. Ninety-six participants reported using good-quality headphones, with the remaining using laptop speakers. Participants’ preferences for the genres used in the study were comparable across the three genres: funk (*M* = 3.37, SD = 1.06), jazz (*M* = 3.13, SD = 1.41), and rock (*M* = 3.42, SD = 1.36), with no statistically significant differences between means. Participants received £6.50 per half hour, with a median completion time of 19 min. The study was approved by the University of Birmingham Research Ethics Committee.

### Stimuli

Twelve stimuli were selected from a study by Senn et al. (32), which collected groove ratings for real music. We hoped real music would maximize the embodied pleasure in the experiment, compared with artificial stimuli. We chose four tracks for each of three different conditions of music, based on both genre and groove ratings collected in Senn et al.'s study—funk with high groove ratings, jazz funk with medium groove ratings, and rock with low groove ratings. The jazz funk stimuli are labeled funk in the Senn et al. study, but listening to them indicated they were better described as a fusion of jazz and funk. To further justify this separation between funk and jazz funk, we conducted *t*-tests on Senn et al.'s groove questionnaire ratings. We found that funk was rated significantly higher than jazz funk on the items “I would like to dance to this music,” “I like to listen to this music,” and “This music is great for a party” (all *P*'s < 0.01). We chose not to include the pop genre, which was rated as high groove in Senn et al.'s study, because most of the highly rated songs were well-known hits that would likely be familiar to the participants. All three chosen genres were rated as significantly different in groove ratings in Senn et al.'s study (all *P*'s < 0.05), with funk rated highest, followed by jazz funk and rock, in that order. See Table S1 for track names and average groove ratings from Senn et al.'s study.

We deliberately avoided well-known tracks and selected tracks that we hoped would be relatively unknown. Nonetheless, a linear mixed effects model (see “Statistical analysis” section below for its random structure) showed that the funk tracks were rated as more familiar than the jazz funk tracks (*B* = −0.3819, df = 17.28, *t* = −3.114, *P* = 0.006) but not the rock tracks (*B* = −0.2500, df = 24.55, *t* = −1.850, *P* = 0.076). While the estimated means and mean differences between familiarity ratings for each genre were generally low (means: funk = 2.47, jazz funk = 2.09, rock = 2.22, all SEs ∼0.121), we decided to include familiarity as a covariate in our analysis of ratings and test effects of familiarity on BSMs.

We analyzed the pulse entropy of the stimuli, using the MIR Toolbox mirpulseclarity function, specified with the “EntropyAutocor” setting (30). Jazz funk had the highest level of pulse entropy (mean = 0.750, SD = 0.030), followed by funk (mean = 0.734, SD = 0.028) and rock (mean = 0.722, SD = 0.039), in that order, although *t*-tests showed no statistically significant differences between them. The overall range was 0.669–0.787, which roughly falls within a normal range of real music studied in groove research (31).

### Procedure

**Fig. 1.**
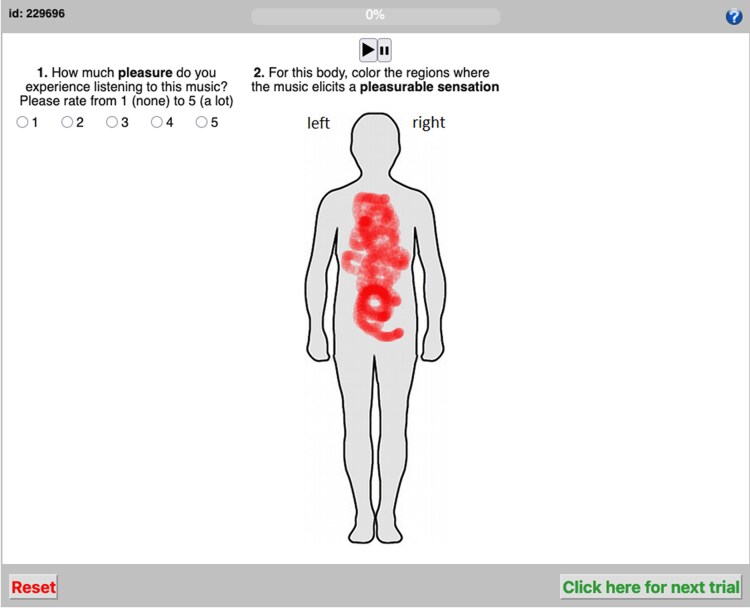
The task was to first rate either wanting to move or pleasure in response to the music, and then color on a 2D body silhouette where in the body they felt that sensation. Half of the participants rated wanting to move first, and then pleasure, with the other half in the opposite order.

Participants were recruited through prolific and taken to a separate website hosting the experiment. We adapted the code (in PHP) for the experiment from Nummenmaa et al. (12). Participants first gave informed consent and reported demographic data, including whether they were using high-quality headphones. The experiment was repeated over two blocks, with half of the participants (chosen randomly) responding to questions about wanting to move for the first repetition. They were asked to first rate how much the music made them want to move on a 5-point Likert scale. Then, they were asked to use their computer mouse to color a blank body silhouette to indicate where in the body they experienced wanting to move, coloring as little or as much as they liked. They then rated how familiar they were with the music. Then, they heard the music again in the second block, first rating how much pleasure they experienced listening to the track and coloring where in the body they felt pleasure on the body silhouette (Fig. 1). The second half of the participants completed the blocks in reverse, starting with pleasure and familiarity, and ending with wanting to move. Familiarity was always rated in the first block to gather data during the first exposure to the music. Tracks were randomized within each block. At the end of the final block, participants were asked to rate their preference for the three genres on 5-point Likert scales, as represented by the music they had heard in the study.

### Statistical analysis

Ratings were analyzed in RStudio (version 3.6.3). The two groove components of wanting to move and pleasure were analyzed separately. We first investigated the similarity between ratings within each genre based on the Euclidean distance in order to test the suitability of using genre as a way to induce different levels of groove. Then, we conducted linear mixed effects models, with genre as the fixed effect, familiarity and formal and informal musical training as fixed covariates, and with by-participant and by-stimulus random intercepts and slopes for the effects of genre (46). We included the interactions of genre with musical training. Funk was set as the “treatment” variable, and as a result, contrast coefficients for genre represent funk > jazz funk and funk > rock only. In the post hoc comparisons, all genres were compared with each other.

All BSM data were analyzed in Matlab (version 2023a), using adapted scripts from Nummenmaa et al. (12). We reconstructed the coloring patterns for each participant and each trial from the computer mouse data (time, location, and brush size) during coloring. We then screened for any anomalous coloring patterns of the BSM and removed any data falling outside the body silhouettes.

To determine where in the body participants felt wanting to move and pleasure in response to the music, we first performed mass univariate t tests across subjects’ BSM for each pixel, comparing wanting-to-move and pleasure responses for each genre against zero, with *t*-values corrected for multiple comparisons using the false discovery rate (FDR) method. We then conducted repeated-measures *F* tests for each pixel, producing BSMs with color maps for each variable, groove component (wanting to move and pleasure), and genre (funk, jazz funk, and rock), as well as the interaction. *F* statistics were FDR corrected for multiple comparisons. We then performed post hoc t tests across all pixels to compare the different levels for each condition. *T* values were corrected for multiple comparisons using the FDR method. For all BSMs, the color intensity of the heat maps represents the magnitude of the statistic (*F* or *t*), with the *t* statistics in the post hoc tests also showing the direction of difference (blue vs. red).

To test the effects of familiarity on BSMs, we calculated the average familiarity across all subjects for each stimulus and then grouped the stimuli into two categories of familiarity: low and high. We then conducted repeated-measures *F* and FDR-corrected post hoc t tests on BSM data by computing *F* and *t* maps of effects on body sensations. We included the groove component as an additional factor and tested the familiarity-by-groove component interaction as well.

When exploring effects of pulse entropy, we first regressed pulse entropy scores against participants’ ratings of the two groove components separately, using linear mixed effects models with formal and informal musical training as additional fixed effects and by-participant and by-stimulus random intercepts. We conducted an additional analysis to test the interaction between pulse entropy and genre on ratings (reported in Supplementary Materials). For the BSM analysis, the effect of pulse entropy was tested by splitting the data into three levels of entropy (low, medium, and high) and computing *F* and FDR-corrected *t* maps, including the interaction with the groove component.

## Results

### Wanting-to-move and pleasure ratings in response to genre

**Fig. 2.**
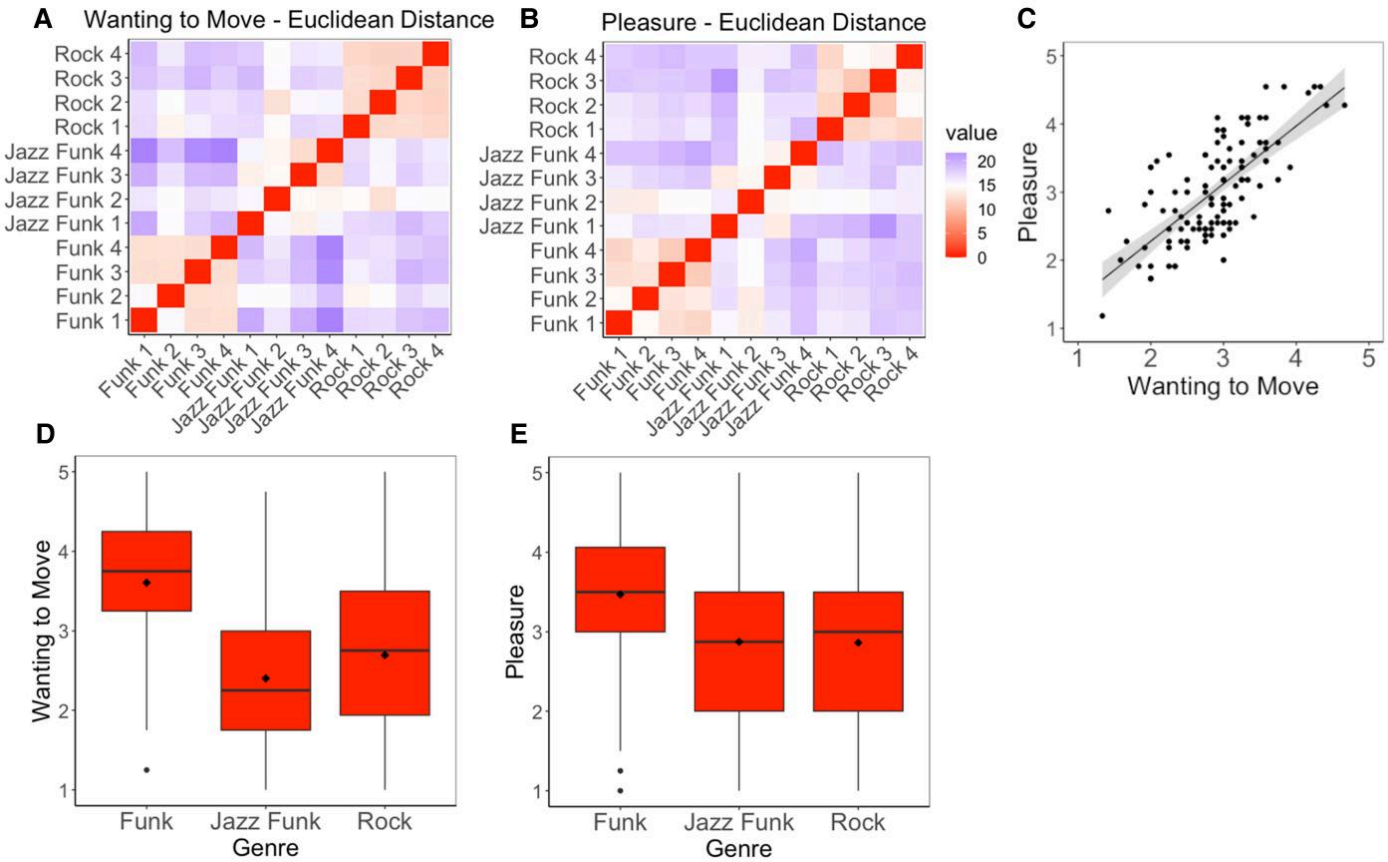
A) Euclidean distance similarity matrices of each stimulus for wanting-to-move ratings. B) Euclidean distance similarity matrices of each stimulus for pleasure ratings. Redder colors indicate more similarity (i.e. less Euclidean distance). C) Correlation between participants’ wanting-to-move and pleasure ratings, averaged across all stimuli. D) Effects of genre on ratings of wanting to move. E) Effects of genre on ratings of pleasure.

We first investigated whether the tracks within the three genres were similarly rated, indicating that they were well suited to represent the genres selected. Euclidean distance similarity matrices are plotted in Fig. 2A and B, showing that ratings were similar within funk and rock. There was overall less similarity in ratings for jazz funk, especially in the pleasure ratings. Compared with the other jazz funk tracks, one track (jazz funk 2) was more similar to a rock track (rock 2) for the movement ratings and more similar to funk tracks (funks 1 and 2) for the pleasure ratings. Overall, this suggests that the tracks within each genre represent reasonable groupings, although less so for jazz funk than funk and rock.

Participants’ ratings of the groove components wanting to move and pleasure, averaged across all stimuli, showed a strong positive correlation (*r*  *=* 0.727, *P* = 0.0001, see Fig. 2C) but since we were interested in differences between these components, they are analyzed separately below.

For wanting to move, linear mixed effects models showed significantly improved fit when adding genre (χ^2^(2) = 19.05, *P* < 0.001) and familiarity (χ^2^(1) = 243.74, *P* < 0.001) to an intercept-only model. There were no effects of musical training or significant interactions. Model coefficients are reported in Table 1, showing effects of funk over jazz funk and rock and the positive effect of familiarity. Post hoc tests corrected for multiple comparisons using the multivariate t-method, comparing all genres, showed that funk was rated significantly higher than both jazz funk (*t* = 5.77, *P* < 0.001) and rock (*t* = 4.36, *P* = 0.004), but there was no significant difference between jazz funk and rock (*t* = −1.30, *P* = 0.421 Fig. 2D).

**Table 1.** Effects of genre and familiarity on ratings of wanting to move and pleasure.

	Estimate	SE	df	*t*	*P*
Wanting to move					
Funk—jazz funk	−1.21	0.18	9.92	−5.77	<0.001
Funk—rock	−0.91	0.21	10.02	−4.36	<0.01
Familiarity	0.97	0.04	1,143.47	24.52	<0.001
Pleasure					
Funk—jazz funk	−0.59	0.20	9.00	−3.90	<0.05
Funk—rock	−0.61	0.20	9.00	−3.00	<0.05
Familiarity	0.95	0.04	1,283.00	23.88	<0.001

For pleasure ratings, model fit improved with genre (χ^2^(2) = 9.23, *P* = 0.010) and familiarity (χ^2^(1) = 291.91, *P* < 0.001). Coefficients are reported in Table 1, showing effects of funk over jazz funk and rock, and the positive effect of familiarity. Post hoc tests corrected for multiple comparisons using the multivariate t-method, between all genres, showed that funk was rated significantly higher than rock (*t* = 3.00, *P* = 0.036), and jazz funk (*t* = 3.06, *P* = 0.033), but there was no significant difference between jazz funk and rock (*t* = 0.06, *P* = 0.999; Fig. 2E).

The rating analyses suggest that funk reliably induces high groove, but that there is no clear differentiation between jazz funk and rock, which are associated with similar, medium ratings. This effect goes beyond any effects of familiarity, which were controlled for in the analysis, and any basic preferences for the genres, which were comparable across all three genres (see details in the “Participants” section).

### Maps of groove-induced bodily sensations

**Fig. 3.**
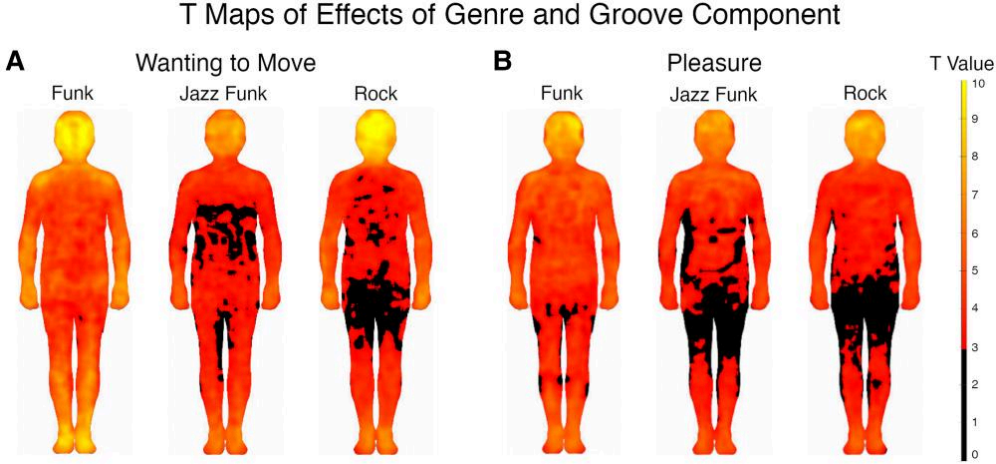
Maps of FDR-corrected mass univariate t tests, comparing body sensations for the two groove components and three genres against zero. A) *T* maps of wanting to move for each genre. B) *T* maps of pleasure for each genre. Regions with higher *t* values indicate increased bodily sensations. Regions in black are below the significant *t* threshold following FDR correction.

While the rating results suggested that jazz funk and rock produced similar wanting-to-move and pleasure ratings, we continue to differentiate these in the BSM analysis, as it may be that they promote different body sensations. The mass univariate t tests for each groove component and each genre are shown as BSMs in Fig. 3. Funk appeared to produce the strongest and most widespread groove sensations. Across both groove components and all three genres, body sensations were strong in the head and extremities. In response to funk, sensations were strong also in the shoulders and hips, especially for wanting to move. Sensations of pleasure were felt also in the chest and abdomen, especially for funk. When listening to rock, head sensations were strong for both groove components, but especially wanting to move.

**Fig. 4.**
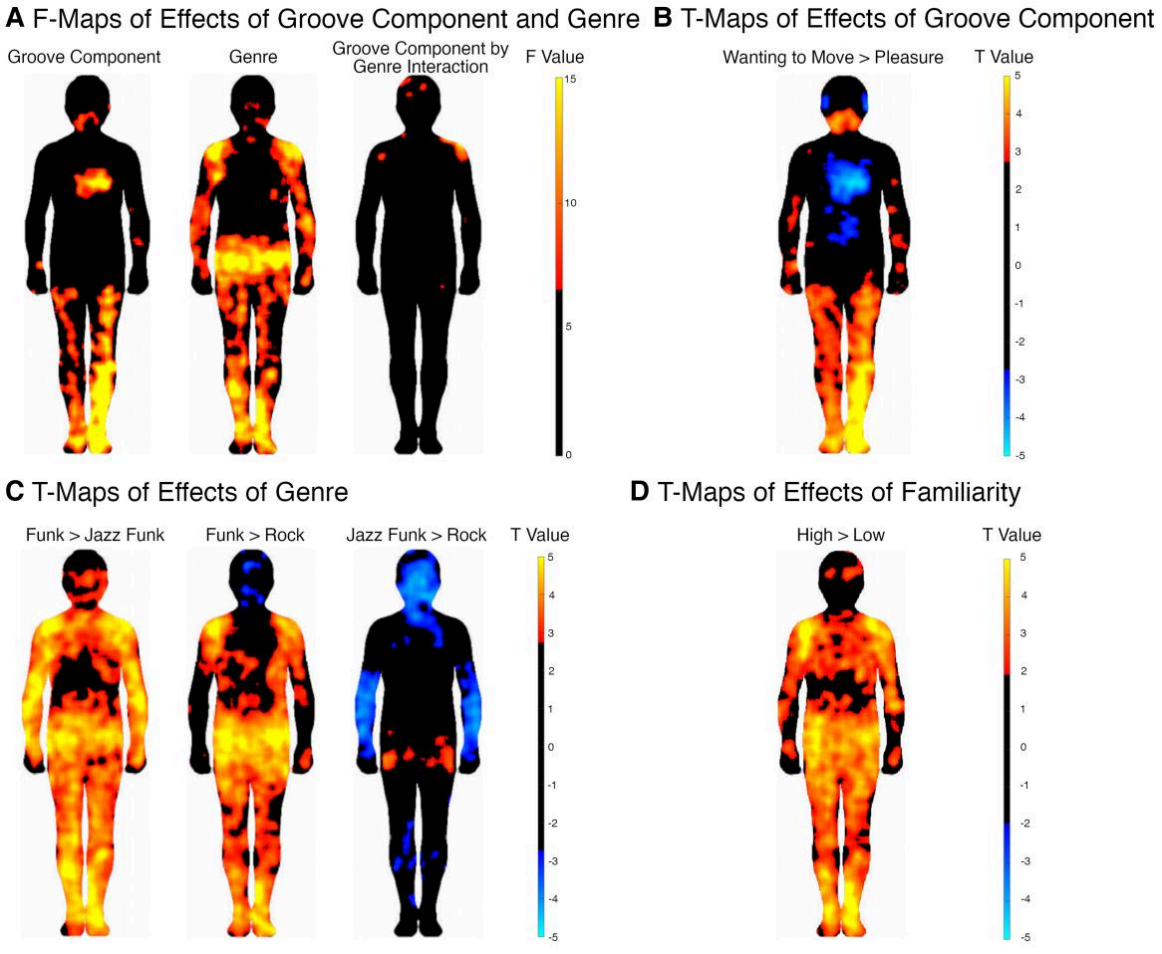
Effects of groove component and genre on FDR-corrected body maps. A) *F* maps of groove component, genre, and their interaction. B) *T* maps for groove component contrast, comparing wanting to move versus pleasure. Regions with positive *T* values (red) indicate increased bodily sensations for wanting to move, while negative *T* values (blue) indicate increased bodily sensations for pleasure. C) *T* maps for genre contrasts, comparing funk, jazz funk, and rock. Regions with positive *T* values (red) indicate increased sensations for the first condition in the contrast pair, and negative values (blue) indicate increased sensations for the second condition in the contrast pair (e.g. in the right-most map of C, red indicates areas where jazz funk produced stronger sensations than rock, and blue where rock produced stronger sensations than jazz funk). D) *T* maps for familiarity contrasts, showing increased sensations in the body for high compared with low familiarity (red). There were no areas where low familiarity was associated with stronger sensations than high familiarity (i.e. no areas in blue).

To investigate any statistically significant differences between these sensations, we conducted an *F* test on BSMs for groove component, genre, and the interaction between these, the results of which are plotted in Fig. 4A. The maps show that there was a significant effect of the groove component in the chest, arms, wrists, legs, feet, and neck/throat/mouth area. For genre, there were significant effects in the head, shoulders, arms, wrists, hips, legs, and feet. A few pixels suggested a significant interaction between these two variables, with some smaller clusters in the head, shoulders, and right hip. We investigated these interactions further to test whether the difference between sensations of the two groove components depended on genre. We found that the increased sensations for wanting to move compared with pleasure in the shoulders and hip were for funk only, while the difference in the head was for jazz funk only (Fig. S2).

Follow-up t tests on BSMs (Fig. 4B) showed that when comparing the two groove components, wanting to move produced stronger bodily sensations in the arms, wrists, legs, feet, and the neck/throat/mouth area (red). Pleasure produced stronger sensations in the chest, abdomen, and ears (blue). For genre (Fig. 4C), we found that funk produced stronger sensations in the shoulders, hips, legs, and feet than both jazz funk and rock. In addition, funk produced stronger sensations than jazz funk in the head, upper chest, abdomen, arms, and hands. Jazz funk produced stronger sensations in the hips than rock, while rock produced stronger sensations in the head than jazz funk and funk. Rock also produced stronger sensations in the arms and legs than jazz funk.

The *F* test on BSM data in response to stimulus familiarity showed effects in the shoulders, arms, hips, legs, and head. The post hoc t tests showed that sensations across almost all parts of the body increased for high compared with low, especially in the shoulders, hips, arms, legs, and feet (Fig. 4D). There was no interaction (blank map) between familiarity and the groove component, suggesting that familiarity affected bodily sensations of wanting to move and pleasure similarly.

### Effects of pulse entropy

**Fig. 5.**
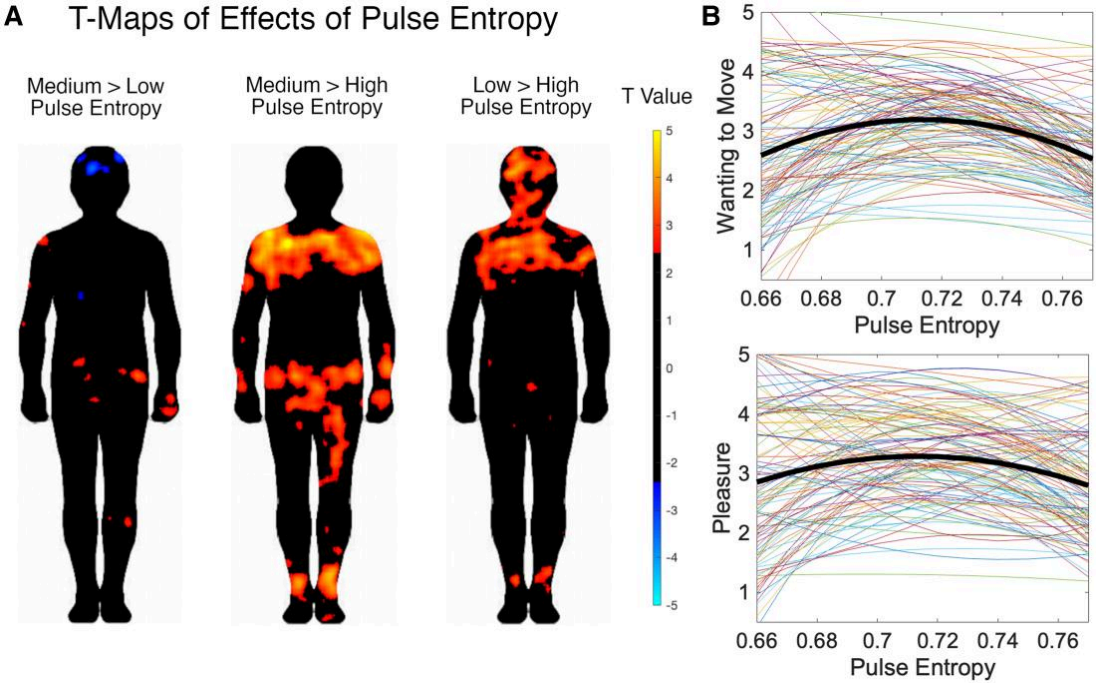
Effects of pulse entropy on FDR-corrected body maps and groove component ratings. A) *T* maps for pulse entropy, comparing low, medium, and high levels, with positive values (red) indicating increased sensations for the first condition in the contrast pair, and negative values (blue) indicating increased sensations for the second condition in the contrast pair (e.g. in the left-most map of A, red indicates areas where medium pulse entropy produced stronger sensations than low pulse entropy, and blue where low pulse entropy produced stronger sensations than medium pulse entropy). B) Effects of pulse entropy on ratings of wanting to move and pleasure.

Our exploratory analysis of pulse entropy on groove component ratings showed that a quadratic effect of pulse entropy (χ^2^(2) = 84.98, *P* < 0.001) and a linear effect of informal musical training (χ^2^(1) = 5.73, *P* = 0.017) improved model fit for wanting-to-move ratings. We report coefficients for both linear and quadratic effects of pulse entropy and the linear effect of musical training (Table 2). Both linear and quadratic effects of pulse entropy were negative and significant, but the quadratic effect was strongest, as indicated by the coefficients. A similar pattern was found for pleasure, with the quadratic effect of pulse entropy (χ^2^(2) = 53.22, *P* < 0.001) and formal musical training improving model fit (χ^2^(1) = 5.19, *P* = 0.023), with significant negative linear and quadratic effects of pulse entropy, the latter slightly stronger than the former (Table 2). Figure 5B plots the quadratic fit for each participant overlaid with the fit for ratings averaged across participants, for both wanting-to-move and pleasure ratings. The analysis of the interaction between pulse entropy and genre showed a complex relationship whereby the curve and range of the U-shape differ depending on the genre (Table S2 and Fig. S1).

**Table 2.** Effects of pulse entropy on ratings of wanting to move and pleasure.

	Estimate	SE	df	*t*	*P*
Wanting to move					
Pulse entropy—linear	−7.24	1.35	716.72	−5.35	<0.001
Pulse entropy—quadratic	−12.82	1.57	274.50	−8.17	<0.001
Informal musical training	0.04	0.01	107.46	2.54	0.012
Pleasure					
Pulse entropy—linear	−4.73	1.29	690.50	−3.68	<0.001
Pulse entropy—quadratic	−9.98	1.49	257.80	−6.70	<0.001
Formal musical training	0.04	0.02	107.58	2.30	0.023

The *F* test on BSM data in response to pulse entropy showed effects on sensations in the upper chest and shoulders, with some smaller clusters also in the hips, ankles, and head. There was no interaction between pulse entropy and the groove component (blank map). The follow-up t test maps (Fig. 5A) showed that medium pulse entropy produced stronger sensations than high-pulse entropy in the upper chest and shoulders, hips, legs, arms, and feet. Low pulse entropy, in turn, was associated with stronger sensations in the head, upper chest, right shoulder and ankles compared with high-pulse entropy. When comparing low and medium pulse entropy, there were a few small, low-intensity clusters showing that low complexity increased sensations in the head and medium complexity increased sensations in the hips, left shoulder, and right arm and leg.

## Discussion

Using ratings and BSMs, this study found that groove is felt in the body, and listeners feel different aspects of the groove sensation in different parts of the body. We found that funk, which was associated with high pleasure and wanting-to-move ratings, increased sensations across the whole body, including the head, shoulders, upper chest, abdomen, arms, hands, hips, legs, and feet. Jazz funk and rock were both rated as eliciting medium levels of wanting to move and pleasure but could nonetheless be distinguished by their body sensations, with jazz funk producing sensations in the hips compared with rock producing sensations in the head and arms. When comparing the two groove components, wanting to move was associated with increased sensations in the arms, wrists, legs and feet, and in the neck/throat/mouth area, while pleasure was associated with increased sensations in the chest and abdomen. Pleasure and wanting-to-move ratings showed an inverted U-shaped relationship with the pulse entropy of the stimuli, and intermediate pulse entropy levels were associated with sensations in the upper chest, shoulders, hips, and ankles. Overall, there appear to be clear patterns of embodied differentiation for different levels and components of groove.

The similarity analyses on pleasure and wanting-to-move ratings suggested that ratings were similar within each genre, especially for funk and rock, although less so for jazz funk. Overall, this confirms that genre can be used to induce different levels of groove, at least when comparing funk to rock. Funk was also rated higher in wanting to move and pleasure compared with both jazz funk and rock. These effects were over and above any effects of familiarity, which affected both ratings positively. The results are generally in support of our first hypothesis, that funk would produce the highest pleasure and wanting-to-move ratings. However, we found no rating differences between jazz funk and rock. Data from an earlier study using the same stimuli (32) suggested that there would be significant differences between all three genres, with funk the highest, followed by jazz funk and rock, in that order. However, in that study, participants were not asked about wanting to move or pleasure directly but rather were asked about their liking of the music and its suitability for dancing and parties (32), potentially explaining the slightly different effects.

We replicated the inverted U-shaped relationship between rhythmic complexity and ratings of wanting to move and pleasure (23–26), where rhythmic complexity was indexed by the music's pulse entropy (31). This supports our fourth hypothesis and the theory that psychologically, groove is engendered by intermediate levels of complexity. Since pulse entropy is a measure of uncertainty regarding the timing of the beat, this aligns with the theory that groove is promoted by strongly weighted prediction errors in the brain (20).

However, pulse entropy did not map directly onto genre, as there were no significant mean differences in pulse entropy between each genre level. In the Supplementary Materials, we report the relationship between genre and pulse entropy on pleasure and wanting-to-move ratings, showing that the curve and range of the U-shape depend on the genre type. It may be that genres reflect other components of groove that were not measured in this study, such as genre associations, immersion, and sociocultural behaviors (5), and these interacted with genre in a different way than pulse entropy, leading to the complex relationship in their effect on ratings. Future research should investigate the relationship between rhythmic complexity and genre in groove more explicitly.

We also found support for our second and fifth hypotheses that groove would be associated with stronger sensations in both extremities and the chest and abdomen areas of the body. Funk and medium levels of pulse entropy—which produced the highest ratings of wanting to move and pleasure—both elicited the strongest sensations in the chest, shoulders, hips, arms, hands, and legs (although sensations in the chest were only significantly stronger for medium compared with high complexity, not compared with low). Funk also elicited strongest sensations in the head, abdomen, and hands. It should be noted that the clusters in the chest and abdomen were only significantly higher for funk in the *T* maps, not the interaction *F* map, and thus, these results should be interpreted with some caution. The results align with previous research showing that happy and danceable music elicit body sensations and movements in the extremities, including arms, legs, and head (16, 17). We add to this by showing sensations also in the chest and abdomen. These areas, as parts of the torso, also move when dancing and synchronizing to music associated with groove, although with less degrees of freedom than the extremities (34), which might explain the slightly weaker statistical results for these areas.

The torso not only moves to music, but it is also the site of sensations of positive valence in music, specifically in the chest region (16). Furthermore, people experience increased sensations in chest and abdominal regions in response to uncertainty and surprise in harmonic chord progressions (18). Surprise generally is also associated with chest sensations (12). Since uncertainty and surprise are both affective responses to prediction and prediction error (47, 48), this has led to the suggestion that these areas are where the embodied dimensions of predictive processing are felt (18). The findings from our exploratory analysis support this by showing that intermediate levels of pulse entropy, which are thought to stimulate the predictive processing of groove (31), are associated with upper chest sensations. However, this effect is only significantly different when compared with high levels of pulse entropy, and not low. Both medium and low pulse entropy elicited stronger sensations in the upper chest compared with high-pulse entropy. It may be that the real music used in this study did not represent low enough levels of pulse entropy to show a big enough differentiation in the core body sensations, although the overall range of entropy levels was comparable with other studies focusing on groove (31).

Until now, it has not been clear whether the two components of groove studied here—movement and pleasure—are part of the same cognitive or affective construct or whether they can be functionally differentiated (43, 45). In groove, they are at least bidirectionally linked, as movement can produce pleasure, and pleasure can invite movement, possibly via a hedonic feedback loop (33). However, our data here suggest that as an embodied feeling, there are qualitative differences between these two components of groove. The core parts of the body, including both chest and abdomen, were more strongly associated with the pleasure component of groove, and the extremities were more strongly associated with wanting to move, supporting our third hypothesis. The two dimensions of groove can broadly be distinguished by their association with either skeletomuscular (movement) or somatovisceral (pleasure) sensations.

Nummenmaa et al. (12) showed that both positive and negative emotions were associated with sensations in both the chest and abdomen and the extremities, suggesting that they likely reflect a combination of skeletomuscular and somatovisceral sensations. For example, anger was associated with strong sensations in the arms (skeletomuscular) and chest (somatovisceral). However, the emotions studied by Nummenmaa et al. (12) could be considered more complex compared with the more reward-oriented feelings studied here. In music, anticipation (wanting) and experience (liking/pleasure) of reward have been associated with distinct brain activations in the caudate and nucleus accumbens, respectively (49). For groove, wanting to move activates putamen and medial orbital frontal areas, while pleasure is more related to nucleus accumbens activity (37). Here, we show that, at least in groove, distinctions can also be made between the skeletomuscular wanting and somatovisceral liking of musical reward.

These sensations are part of the interoceptive dimension of embodied emotions (15) and the proprioceptive dimension of motor processes, which have both been modeled as the active inference component of predictive processing (50, 51). That is, emotions and movements are thought to reflect predictions and prediction errors regarding our perceived bodily sensations. Therefore, groove may be linked to predictions relating to both somatovisceral sensations (e.g. interoceptive feedback from the heart and lungs) and skeletomuscular sensations (e.g. proprioceptive feedback from moving along to the beat).

We explored the role of temporal prediction in groove by testing effects of pulse entropy, which is a measure of beat timing uncertainty. Learning, via the reduction of prediction errors and uncertainty over time, is thought to be intrinsically rewarding (52). Uncertainty resulting from medium rhythmic complexity potentiates maximal learning and thus reward (20). Moving along to these rhythms can reinforce the meter and beat, thus increasing the rate of learning. Therefore, the predicted interoceptive and somatosensory feedback from the body resulting from covert or simulated movements to the beat may be the locus of reward associated with such prediction-based learning. This hypothesis is potentially supported by our finding that more and larger clusters resulted from contrasting medium- versus high-pulse entropy compared with low- versus high-pulse entropy, suggesting that medium pulse entropy leads to a more widespread somatosensory network of embodied prediction-based learning. The precise role of such a network in the predictive processing of music needs further research.

This suggestion also poses a potential limitation in the study: whether participants only *felt* wanting to move or whether they actually *moved* the indicated body regions when listening to the music. This includes singing, which could explain the cluster of sensations reported in the neck/throat/mouth area when rating wanting to move to the music. We did not instruct participants not to move or sing, as this could be experienced as restrictive and was difficult to monitor due to the online nature of the experiment. If participants did move or sing in response to their feelings of wanting to move, then it may be that the embodied experience of groove might be better explained through proprioception rather than interoception (53).

While we aimed for the music in our experiment to be unfamiliar, and the average ratings of familiarity for all the tracks were generally low (range of 1.89–2.64, on a 1–5 point scale), we did find an effect of familiarity on ratings of wanting to move and pleasure, in alignment with previous research (32). However, the effect of familiarity did not cancel out the effects of genre, which still significantly affected ratings. We also found that familiarity affected how strongly groove was felt in the body. Highly familiar music led to stronger groove sensations across almost all parts of the body, especially in the shoulders, hips, arms, legs and feet. The effect of familiarity did not interact with groove component, meaning that familiarity affected wanting-to-move and pleasure sensations in the body similarly. Combined, these results suggest that familiarity may increase groove sensations, both as subjective feelings and sensations in the body. It should be noted that our BSM analyses were based on stimulus familiarity ratings averaged across participants and thus might be best understood as a measure of “popularity” rather than subjective familiarity. More systematic study of familiarity should help shed further light on its effects on body sensations of groove in the future.

In addition, there are likely further dimensions of groove that contribute to embodied feeling than those studied here. For example, the polyrhythms of funk have been associated with a kinesthetic epistemology (6) and the syncopated rhythms of electronic dance music with the social distribution of affective atmosphere (54). In other words, the embodiment of groove goes beyond the mere body movements and visceral sensations that it elicits. Future empirical research should address the embodiment of groove in a more holistic way.

We conclude that groove is felt in the body and different genres and groove's rhythmic complexity and predictability affect where in the body sensations are felt. The movement and pleasure components of groove elicit sensations in different parts of the body, with the former felt more in the extremities and the latter more in the chest and abdomen. Our results broadly support the suggestion that the affective dimension of musical predictions is felt in the chest and abdomen, adding to theories that predictive processing is fundamentally embodied.

## Supplementary Material

pgaf306_Supplementary_Data

## Data Availability

All data, scripts, and materials are available at https://osf.io/nqm7p/.
