# Supplementary material for: Body maps of the sensation of musical groove
Source: PNAS Nexus. 2025 Sep 24;4(10):pgaf306. doi: 10.1093/pnasnexus/pgaf306 (PMC12503160; doi:10.1093/pnasnexus/pgaf306)
Supplement: pgaf306_Supplementary_Data [file pgaf306_supplementary_data.pdf]

Supplementary Materials

BODY MAPS OF THE SENSATION OF MUSICAL GROOVE

Maria A. G. Witek[1], Tomas E. Matthews[2], Toni A. Bechtold[1,3] and Virginia Penhune[4]

[1]Department of Music, University of Birmingham, UK
[2]Center for Music in the Brain, Aarhus University, Denmark
[3]Lucerne School of Music, Lucerne University of Applied Sciences and Arts, Switzerland
[4]Department of Psychology, Concordia University, Canada

Stimulus selection

Table S1: Stimuli used and average groove ratings from Senn et al. 2019

Stim No.	Stim name	Track and artist name	Assigned Genre	Groove rating	Avg. groove rating by genre
1	JacA_3	"Do the funky chicken" Rufus Thomas	Funk	0.87	0.74
2	JohR_4	"Shining star" Earth, Wind & Fire	Funk	0.75	
3	DeiA_2	"Lettsanity" Lettuce	Funk	0.73	
4	ErrG_3	"Sing a simple song" Sly & The Family Stone	Funk	0.61	
5	GroD_5	"Alone & easy target" Foo Fighters	Rock	-0.77	-0.91
6	WarB_3	"Psycho man" Black Sabbath	Rock	-0.87	
7	PeaN_2	"Far cry" Rush	Rock	-0.87	
8	PorM_5	"Caught in a web" Dream Theater	Rock	-1.13	
9	PhiS_3	"Soothsayer" Simon Phillips	Jazz Funk	-0.32	-0.45
10	SteB_3	"Pegasus" Larry Goldings	Jazz Funk	-0.46	
11	HakO_1	"Techno" John Scofield	Jazz Funk	-0.51	
12	BroG_4	"Summer madness" Kool & The Gang	Jazz Funk	-0.51	

Senn O, Bechtold TA, Hoesl F, Kilchenmann L. Taste and familiarity affect the experience of groove in popular music. Musicae Scientiae. 2021, 25(1):45–66, https://doi.org/10.1177/1029864919839172

Interaction between pulse entropy and genre on ratings of wanting to move and pleasure

Since wanting to move and pleasure ratings were affected both by genre and pulse entropy, separately, but there was no overall difference in pulse entropy between the different genres, we decided to conduct an additional analysis investigating the interaction between pulse entropy and genre on these ratings. We specified a linear mixed effects model with the interaction between the quadratic effect of pulse entropy and genre, along with their main effects, as fixed effects, and by-participant and by-stimulus random intercepts. The results showed that, for wanting to move, the interaction added significantly to an intercept only model ($\chi^2(8) = 322.72$, $p < .001$). The coefficients are reported in table S2. Since the main effects of pulse entropy and genre are reported in the main text, we only report the coefficients for the interactions here, which indicate that the quadratic slopes of pulse entropy were significantly different for funk compared to jazz funk and for funk compared to rock. This is plotted in Figure S1 (left panel), which indicates that rock and jazz funk together make a clear inverted U-shape, with rock at the lower end and jazz funk at the higher end of pulse entropy, with some overlap in the middle. Funk occupies the middle range of entropy

and is rated the highest on wanting to move, but also has a slight (non-inverted) U-shaped slope on its own. For pleasure, the interaction also added significantly to the model fit ($\chi^2(8)$ =136.48, $p < .001$). The coefficients in Table S2 show that the linear slopes differed for funk compared to jazz funk and rock. The plot in Figure S1 (right panel) shows a similar pattern as for wanting to move, but with a less pronounced inverted U-shape represented by rock and jazz funk, and pleasure ratings for funk being closer to the ratings for the other genres. The overlap in ranges of pulse entropy between the three genres might explain why there were no significant differences in mean pulse entropy across the three genres (see methods of the main paper).

Table S2 Effects of pulse entropy-by-genre interaction on ratings of wanting to move and pleasure

Wanting to Move	*Estimate*	*SE*	*df*	*t*	*p*
Linear effect – funk > jazz funk	6.14	10.14	8.98	0.60	0.560
Quadratic effect – funk > jazz funk	-37.16	11.25	7.27	-3.30	0.012*
Linear effect – funk > rock	2.16	3.41	1105.75	0.63	0.526
Quadratic effect – funk > rock	-23.22	7.34	53.13	-3.16	0.003*
Pleasure					
Linear effect – funk > jazz funk	-21.27	8.45	32.64	-2.52	0.017*
Quadratic effect – funk > jazz funk	-4.93	8.78	11.37	-0.56	0.585
Linear effect – funk > rock	-7.50	3.44	1095.52	-2.18	0.029*
Quadratic effect – funk > rock	-8.84	6.65	44.77	-1.33	0.191

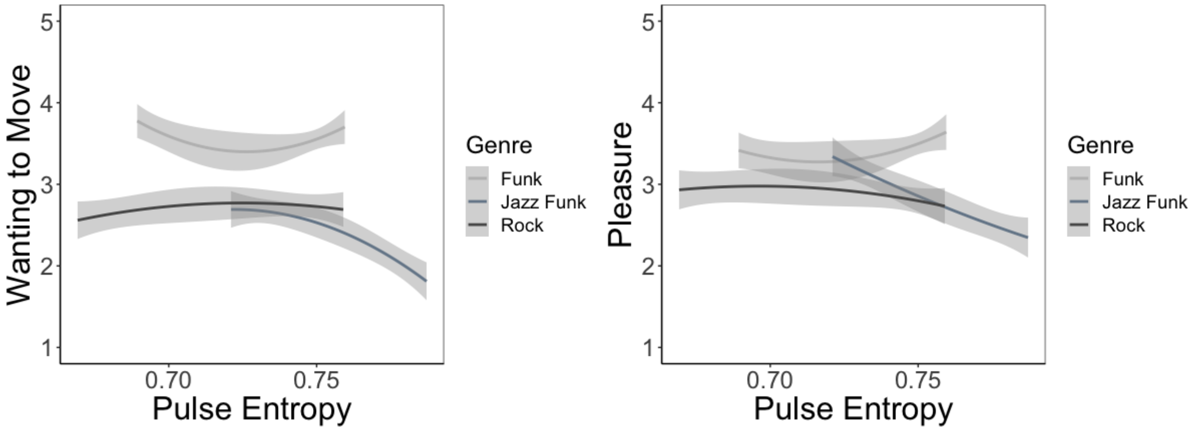

Figure S1. Interaction between pulse entropy and genre on ratings of wanting to move and pleasure.

Interaction between groove component and genre on body sensation maps (BSMs).

To further investigate the areas of significance shown in the F-maps of the interaction between groove component and genre (Figure 4A in the main text), we plot the t-maps contrasting wanting to move against pleasure for each genre separately, in Figure S2. These plots suggest that the interaction effects found in the shoulder and hip are a result of the stronger sensations of wanting to move than pleasure in these areas for funk only. The interaction effect found in the head is a result of stronger pleasure sensations than wanting to move for jazz funk. There were some additional clusters in the chest and abdomen for jazz funk and rock, showing that sensations in these areas for these genres were stronger for pleasure than wanting to move. The lack of this cluster for funk suggests that sensations in

these areas were similar for wanting to move and pleasure. Furthermore, there were some clusters in the legs of all genres, suggesting that sensations here were stronger for wanting to move than pleasure. However as these additional clusters were not significant in the main F-test interaction (in Figure 4A in the main text), these findings should be interpreted with caution.

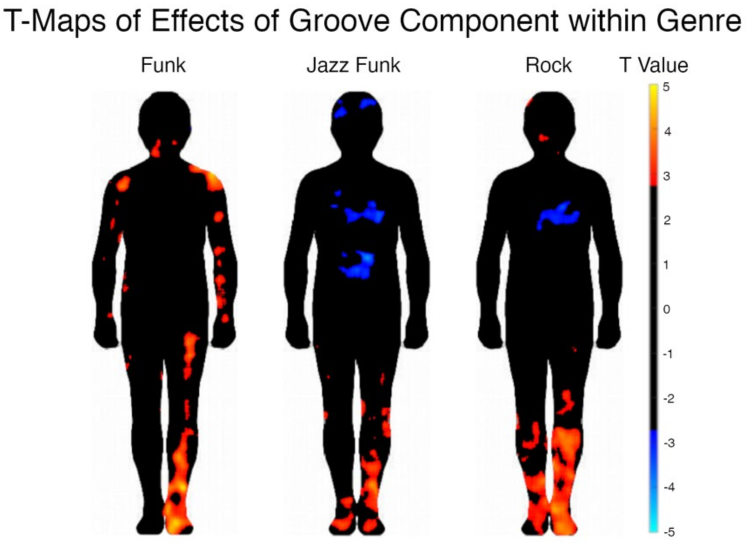

Figure S2. Maps of FDR-corrected T-contrasts comparing groove component—wanting to move - pleasure—within each genre. Positive values (red) indicate increased sensations for wanting to move and negative values (blue) indicate increased sensations for pleasure.
